# Nitric oxide signaling in adipose triglyceride lipase-deficient microvascular endothelial cells

**DOI:** 10.1186/2050-6511-13-S1-A22

**Published:** 2012-09-17

**Authors:** Marion Mussbacher, Sarah Winkler, Günter Hämmerle, Rudolf Zechner, Bernd Mayer, Astrid Schrammel

**Affiliations:** 1 Department of Pharmacology and Toxicology, Karl-Franzens University Graz, 8010 Graz, Austria; 2 Department of Molecular Biosciences, Karl-Franzens University Graz, 8010 Graz, Austria

## Background

Adipose triglyceride lipase (ATGL) has been characterized as key enzyme of mammalian triglyceride catabolism. Mice with global ATGL deficiency were previously described to suffer from lethal cardiac dysfunction that originates from defective peroxisome proliferator-activated receptor alpha (PPARα) signaling in the heart. Experiments from our laboratory demonstrated that endothelium-dependent micro- and macrovascular relaxation is severely blunted in those mice. The aim of the present study was to investigate this phenomenon on a cellular level.

## Methods and Results

Microvascular endothelial cells were isolated from hearts of wild-type (WT) and ATGL(−/−) mice and immortalized to create WT-MyEnd and ATGL(−/−)-MyEnd cells, respectively. Cells were cultured to passage 2–4 and characterized for different parameters of PPARα and NO signaling. Real-time PCR analysis revealed that PPARα mRNA expression was reduced more than 50% in ATGL(−/−)-MyEnd cells, which reflects well the situation in ATGL-deficient hearts. By contrast, mRNA expression of peroxisome proliferator-activated receptor-gamma coactivator (PGC-1α) was similar in WT and ATGL-deficient cells. Likewise, mRNA levels of different PPARα target genes were unaffected by knockout of ATGL. Protein expression and activity of endothelial nitric oxide synthase (eNOS) were measured by Western blot and conversion of L-[^3^H]arginine into L-[^3^H]citrulline, respectively. Both parameters were almost identical in WT-MyEnd and ATGL(−/−)-MyEnd cells. To investigate if accelerated breakdown of NO due to increased formation of reactive oxygen species (ROS) occurs in ATGL-deficient endothelial cells, we measured mRNA and protein expression of xanthine oxidase (XO) and NADPH oxidase isoforms NOX2 and NOX4. However, no differences in mRNA or protein expression were observed. A similar result was achieved in experiments measuring ROS formating in homogenates of WT and ATGL-deficient cells using lucigenin-enhanced chemiluminescence.

## Conclusions

Our results indicate that, albeit impaired PPARα, NO signaling and bioavailability are not compromised by ATGL deficiency in microvascular endothelial cells. Currently, other mechanisms responsible for the observed endothelial dysfunction are investigated in our laboratory.

